# Cervical Cancer Precursors and Hormonal Contraceptive Use in HIV-Positive Women: Application of a Causal Model and Semi-Parametric Estimation Methods

**DOI:** 10.1371/journal.pone.0101090

**Published:** 2014-06-30

**Authors:** Hannah H. Leslie, Deborah A. Karasek, Laura F. Harris, Emily Chang, Naila Abdulrahim, May Maloba, Megan J. Huchko

**Affiliations:** 1 Division of Epidemiology, University of California, Berkeley, California, United States of America; 2 Joint Medical Program, University of California, Berkeley, and University of California San Francisco, San Francisco, California, United States of America; 3 Pulmonary Medicine, University of California San Francisco, San Francisco, California, United States of America; 4 Center for Microbiology Research, Kenya Medical Research Institute, Nairobi, Kenya; 5 FACES, Family AIDS Care and Education Services, Kisumu, Kenya; 6 Department of Obstetrics, Gynecology and Reproductive Sciences, University of California San Francisco, San Francisco, California, United States of America; State University of Maringá/Universidade Estadual de Maringá, Brazil; Consolaro

## Abstract

**Objective:**

To demonstrate the application of causal inference methods to observational data in the obstetrics and gynecology field, particularly causal modeling and semi-parametric estimation.

**Background:**

Human immunodeficiency virus (HIV)-positive women are at increased risk for cervical cancer and its treatable precursors. Determining whether potential risk factors such as hormonal contraception are true causes is critical for informing public health strategies as longevity increases among HIV-positive women in developing countries.

**Methods:**

We developed a causal model of the factors related to combined oral contraceptive (COC) use and cervical intraepithelial neoplasia 2 or greater (CIN2+) and modified the model to fit the observed data, drawn from women in a cervical cancer screening program at HIV clinics in Kenya. Assumptions required for substantiation of a causal relationship were assessed. We estimated the population-level association using semi-parametric methods: g-computation, inverse probability of treatment weighting, and targeted maximum likelihood estimation.

**Results:**

We identified 2 plausible causal paths from COC use to CIN2+: via HPV infection and via increased disease progression. Study data enabled estimation of the latter only with strong assumptions of no unmeasured confounding. Of 2,519 women under 50 screened per protocol, 219 (8.7%) were diagnosed with CIN2+. Marginal modeling suggested a 2.9% (95% confidence interval 0.1%, 6.9%) increase in prevalence of CIN2+ if all women under 50 were exposed to COC; the significance of this association was sensitive to method of estimation and exposure misclassification.

**Conclusion:**

Use of causal modeling enabled clear representation of the causal relationship of interest and the assumptions required to estimate that relationship from the observed data. Semi-parametric estimation methods provided flexibility and reduced reliance on correct model form. Although selected results suggest an increased prevalence of CIN2+ associated with COC, evidence is insufficient to conclude causality. Priority areas for future studies to better satisfy causal criteria are identified.

## Introduction

Cervical cancer is the third most common cancer among women worldwide; 85% of the global burden is in developing countries [1]. An important, unanswered question in the field of cervical cancer prevention is whether use of combined oral contraceptives (COC) – pills that contain both estrogen and progesterone - increases cervical cancer risk [2], [3]. While the risk of many other cancers is lower in COC users than non-users, cervical cancer rates are generally higher among COC users in observational studies [4]. Systematic reviews of observational epidemiologic studies have found an association between cervical cancer risk and use of COC, particularly for increasing duration of COC use and for current and recent use rather than use in the distant past [2], [5]. The International Agency for Research on Cancer has named COC a carcinogen in part because of the relationship between COC and cervical cancer [6].

Hypotheses of a biologic basis for this association include a relationship between COCs and increased vulnerability to human papillomavirus (HPV) infection, with or without subsequent promotion of abnormal cell proliferation. Existing studies do not show a difference in HPV prevalence between COC users and non-users [7]. However, animal models and in vitro studies suggest that estrogen and progestin could affect gene expression of HPV to stimulate cell proliferation in the human cervix [6]. Evidence is suggestive but by no means conclusive of a causal relationship: the observed association may be a result of confounding due to behavioral factors, particularly reduced use of barrier protection among women choosing COC. Risk factors for cervical cancer include poverty, family history, early age at first pregnancy, and having 3 or more full-term pregnancies [8]; the complex interplay of biological factors and interconnected behavioral factors such as contraceptive use and pregnancy outcomes renders the isolation of a causal link from COC use to cervical cancer especially challenging.

Identifying an increased risk due to COC is particularly important in HIV-positive women, who experience higher incidence of cervical cancer and its precursors [9]–[11], due in part to immunosuppression reducing clearance of HPV [12]. HIV is associated with a younger age of cancer onset and increased disease progression, including invasive cancer; this association is increasingly apparent in developing countries as highly active antiretroviral therapy (HAART) extends survival among HIV positive individuals [13]. The balance between the known risks of unintended pregnancy and potential increased risks of contraceptive use may differ in settings of high HIV prevalence. The existence of a causal relationship between hormonal contraception, especially injectable contraception, and HIV acquisition and progression is an ongoing debate of great public health importance [14]. Examining the outcome of cervical cancer may practically contribute to the scientific debate about the use of COCs in areas with high HIV prevalence.

In this instance as in many others, even the best existing data for distinguishing cause from association are observational. Developments in the field of causal inference [15]. particularly the counterfactual framework, use of directed acyclic graphs (DAGs) for modeling causal structures, and semi-parametric estimation approaches strengthen our capacity to disentangle the complex relationship between sexual behavior, concurrent method use, and other demographic characteristics. In this paper, we demonstrate the use of the counterfactual framework and DAGs to frame causal questions, and we apply 3 semi-parametric estimation tools: g computation, inverse probability of treatment weighting (IPTW), and targeted maximum likelihood estimation (TMLE). The formal language of counterfactuals enables definition of the ideal experiment: observation of the outcome in the same individuals in an exposed and an unexposed state, with all else held constant. Framing the question in this way focuses attention on: a) the primary exposure, such as the timing and duration of COC of interest, b) the precise levels exposure might take between groups being compared, including whether women who have never used COC are an appropriate comparison, and c) the type and measurement of the outcome, in this case cervical intraepithelial neoplasia grades 2 and higher (CIN2+) [16]. Posing the question of interest in the language of counterfactuals sets the framework for identification of the particular target parameter of interest, whether that might be incidence or prevalence, risk or rate. A motivating interest in disease etiology, as in this case on the causes of CIN2+, suggest a parameter on the additive scale, such as a risk difference or prevalence difference [17]. DAGs visually represent causal relationships and enable the identification of confounders based on established rules; in particular, use of DAGs can reduce the number of variables required for control of confounding to a minimally sufficient set. Figure S1 provides an overview of reading and manipulating DAGs; for a full introduction, see references [18]–[20]. By making explicit the hypothesized relationships of measured and unmeasured covariates to exposure and outcome, DAGs support the assessment of identifiability: whether the target parameter of interest can be validly estimated given the measured confounders. To understand the relationship of COC use and CIN2+, we assess if sufficient confounders are measured to interpret the estimated association as a causal effect.

The definition of a clear causal question and identification of a set of confounders sufficient to isolate the effect of interest (or of the best possible set of observed confounders and the resulting assumptions regarding unmeasured confounding) set the framework for estimation, whether with standard regression or more novel approaches. A range of estimation methods have been developed that enable calculation of average population-level effects with control for confounding and robust inference [21]–[23]. Many such tools incorporate model selection via automated algorithms that rely on cross-validation to prevent model overfitting [24]. Models developed through these procedures reduce risk of bias due to incorrect model form. For this question, the association of COC use and CIN2+ among HIV positive women, appropriate analysis entails correctly modeling a range of biological and behavioral covariates. Factors such as CD4+ cell count and age, among others, are likely to relate to the outcome in a non-linear fashion; including them in a model using the incorrect form can bias the results while using successive model selection undermines valid inference. Automated model selection coupled with theoretically grounded inference provides a rigorous alternative to accommodating such analytic complexity [25].

Despite the conceptual strength and analytic flexibility of these tools, the insights and methods from the causal inference literature are just beginning to appear in routine clinical epidemiologic practice (e.g. reference [26]). We illustrate the use of the counterfactual framework, DAGs, and semi-parametric estimation methods in addressing the question: what is the effect of using hormonal contraceptives on the development of CIN2+ among women with HIV? Applying these tools to an observational dataset demonstrates their utility in providing the least biased estimate possible from the available data and, should that estimate not plausibly represent a causal effect due to remaining bias, illuminating specific gaps to address in future studies.

## Methods

### Ethics statement

The Kenya Medical Research Institute and the University of California, San Francisco institutional review boards provided ethical approval for analysis of this program dataset. Participants did not provide written consent for inclusion in this specific analysis; all patient records were anonymized and de-identified.

### Causal model

Applying the counterfactual framework, we defined the quantity of interest to be the difference in prevalence of CIN2+ due to any exposure to COC compared to no exposure. Alternative questions of interest could incorporate duration and recency of exposure, which have been shown to modify the magnitude of risk [27]. In the absence of scientific consensus on the latent period of CIN2+ in HIV positive women, the step(s) in the oncogenic pathway that are affected by COC use, and the minimum dose and maximum time since use of COC that could result in CIN2+, we elected to use a broad definition of exposure. We developed a DAG by 1) identifying covariates related to COC (exposure) or CIN2+ (outcome), 2) placing these variables in a plausible temporal order, and 3) denoting remaining uncertainty with a node (i.e. variable) labeled ‘U’ for unknown. For example, educational attainment may influence COC use; education serves as a proxy for poverty, which potentially increases CIN2+ risk. Education predates sexual partnership status in this adult population. Uncertainty remains in the relationship of COC to CIN2+ after accounting for education and the other named variables.

We updated the full causal model to reflect the variables measured in the observed data. We subsequently assessed whether the causal relationship of interest could be isolated from the effect of third variables using the measured covariates only by applying the backdoor criterion to identify potential confounding (see Figure S1). The backdoor criterion is equivalent to stating that investigators have identified and measured all common causes of the exposure and outcome as well as of either 1) confounders and exposure or 2) confounders and outcome [20]. If the backdoor criterion holds, controlling for the set of variables that fulfill it enables estimation of a causal effect: confounding can be controlled such that the causal effect is identifiable, a term meaning it is possible to estimate directly from the observed data.

### Data

Data were drawn from a cervical cancer screening program within an HIV care and treatment program in Kisumu, Kenya. Full program procedures and results have been described elsewhere [28]. Women were screened using visual inspection with acetic acid (VIA) alone or in combination with visual inspection with Lugol's iodine (VILI). Positive screening (positive VIA or positive VIA and positive VILI) led to colposcopy and subsequent biopsy to determine treatment. Because only those screening positive could be definitively diagnosed and the adequacy of VIA declines with advancing age [29], only women under 50 were eligible for inclusion in this analysis. All other study data, including clinical and demographic covariates, were drawn from electronic medical records at the HIV clinics and spanned the duration of the individual's enrollment in care; average visit frequency is every 3 months. COC ever-use was defined as reporting oral contraceptive use at any clinic visit prior to cervical cancer screening. Additional covariates extracted from patient data included age, educational attainment, marital status, gravidity, CD4+ cell count nadir, and initiation of highly active antiretroviral therapy (HAART). Missing covariates were imputed using chained multiple imputation [30] to create 10 datasets; estimates were combined across datasets using Rubin's rule. The validity of multiple imputation rests on the assumption of missingness at random (MAR), i.e. that the probability of a variable being observed is random conditional on covariates included in the imputation [30]. MAR is plausible in this context: the primary causes of missing data relate to evolving data quality assurance practices at each site and over time, both of which were accounted for in the imputation.

### Estimation

We implemented 3 methods to estimate the excess prevalence of CIN2+ associated with exposure to COC. Simple substitution (g computation) can be based in parametric regression; it estimates the counterfactual outcome for each observation conditional on covariates to enable the calculation of the population-level difference in prevalence under 2 exposure levels [31]. We fit a logistic regression of CIN2+ on COC, controlling for the covariates identified in the final causal model; we report regression results for comparison to traditional analysis. COC exposure was subsequently set to 1 for the full sample and individual outcomes predicted; repeating this with COC set to 0 provided the population-level prevalence difference. IPTW addresses confounding by modeling the probability of observed exposure status for each individual and weighting observations by the inverse of this probability in an effort to mimic the population that would have been observed if exposure were randomized [21], [32]. For example, a woman whose age, partner status, and pregnancy history make it unlikely that she would take COC (based on the data) but who actually was exposed would be up-weighted substantially. We fit a data adaptive model of COC exposure given covariates, predicted each individual's probability of exposure from the model, and stabilized the estimated weight by the overall probability of being exposed or unexposed. The difference in prevalence between the weighted populations provided the target parameter estimate. TMLE is a more complex method that regresses the outcome on exposure and covariates and then updates this initial estimate using the probability of exposure given covariates to reduce bias for the target parameter [25], [33]. We fit data-adaptive models for both outcome and exposure within TMLE. All analyses were performed in R 3.0 using the TMLE and SuperLearner [24], [34] packages as well as code available from the authors on request.

We implemented each approach for the full dataset and sensitivity analyses restricted to individuals with at least 6 months of observed data and reported COC use at greater than 20% of patient visits. Variance estimates were obtained through bootstrap sampling (200 iterations for each imputed dataset) for g computation and IPTW and from the variance of the influence curve for TMLE. Finally, we assessed potential violations of the positivity assumption (i.e. that there is a non-zero probability of each exposure level for all covariate combinations) by inspecting the distribution of exposure within covariate categories and the symmetry of bootstrap estimates.

## Results

### Causal model

**Figure 1:**
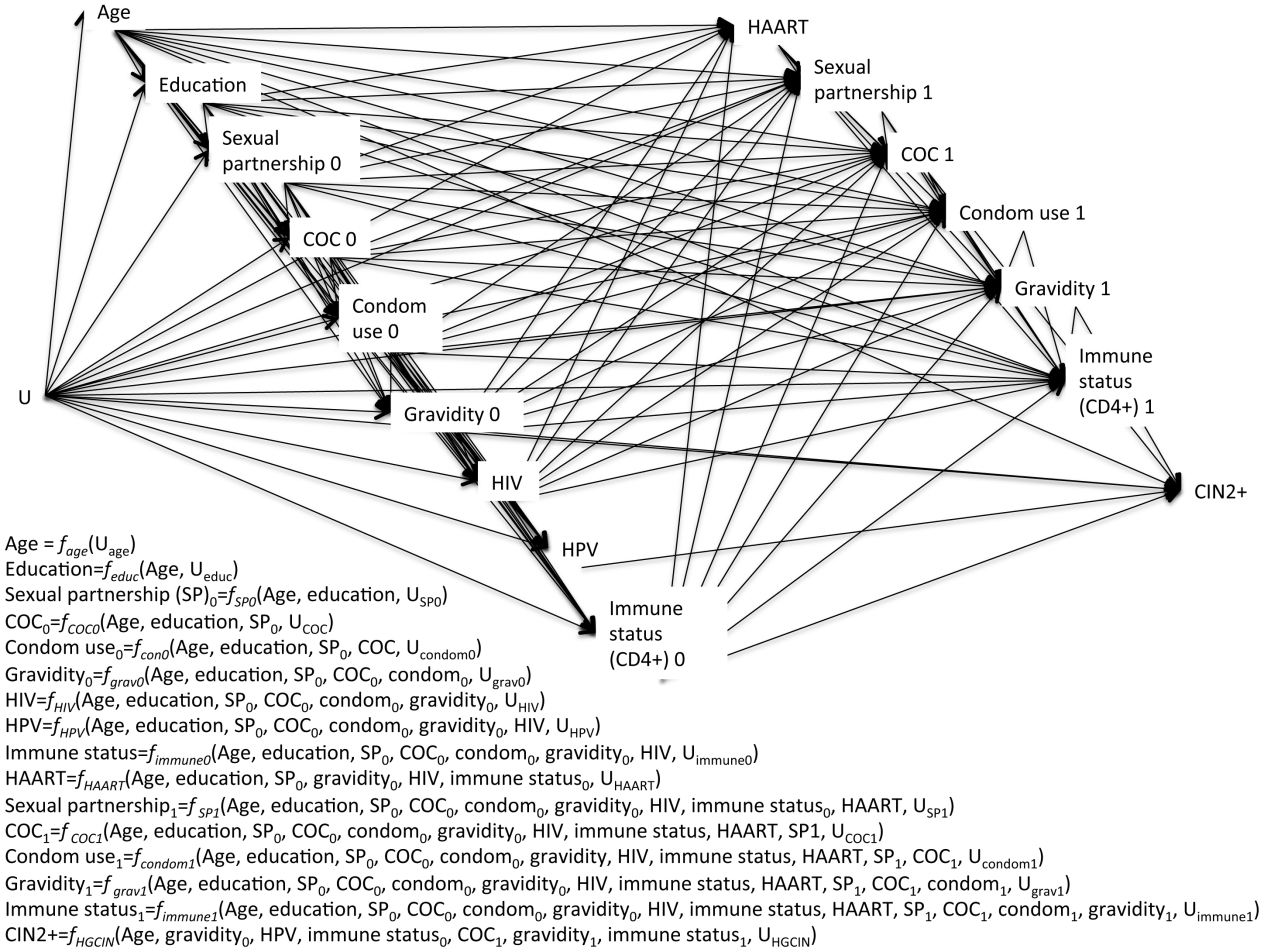
Full causal model in directed acyclic graph and structural causal model forms. Proposed causal model in equivalent graph and formula forms of the relationship between contraception use and cervical pre-cancer over 2 time points. DAG: directed acyclic graph; SCM: structural causal model; SP: sexual partnership; COC: combined oral contraception; HAART: highly active antiretroviral therapy; CIN2+: cervical intraepithelial neoplasia grades II and above; HIV: human immunodeficiency virus; HPV: human papilloma virus; U: unknown.

Figure 1 shows the hypothesized full causal model, showing 2 time points (0, 1), although the relationships shown would iterate over time. The figure includes a translation of the visual DAG presentation into a structural causal model [35] (SCM) using equations; SCMs can be particularly valuable in presenting complex models such as this one. The U node represents unknown causes of all other nodes on the DAG; this can include random chance as well as causal factors. Each connection (path) on the graph represents a potential causal link; any excluded path is a strong assumption of no causal relationship. Representing nodes at multiple time points enables clear depiction of variables that repeatedly affect each other, such as COC use and gravidity. Separating time points in this way ensures that all paths in the model are unidirectional, thus removing potential concerns of reverse causation. We present 2 causal pathways between COC and CIN2+. One, via HPV, represents increased vulnerability to HPV infection. The second, chronologically after HPV infection and oncogenic transformation, depicts enhanced growth of abnormal cells in the presence of COC. Given the complexity of the model, excluded paths can be more clearly read from the SCM equations than the DAG. Briefly, the basis for each exclusion restriction is as follows. We exclude HPV as a cause of subsequent immune status and HAART use due to lack of biological evidence that HPV affects serum CD4+ cell count and hence HIV treatment decisions. We further exclude HPV as a cause of subsequent sexual partnership, COC use, condom use, and gravidity, as undiagnosed HPV infection would be unlikely to affect behavior, and HPV infection prior to development of CIN has not been shown to affect fertility [36]. We assert that COC use and condom use do not cause HAART use as in this setting prescription of HAART is based on CD4+ cell count and presence of opportunistic infections, neither of which is likely to be affected by COC or condom use. Finally, we remove paths from education, sexual partnerships, COC use at time 0, condom use, HIV, and HAART to CIN2+. Many of these variables are indirect causes of CIN2+ through intermediate variables such as HPV or immune status; we suggest there is no direct connection outside of the mediating variables. For example, condom use can cause CIN2+ only via HPV in this model. Aside from these exclusions, all other time-ordered connections are considered plausible and are included in the full causal model.

Laying out the causal structure clarifies a key decision point: can both paths from COC to CIN2+ be estimated? Limitations in the data prevented us from assessing exposure and covariates prior to disease initiation; therefore we focused on the pathway from COC use during HIV care to disease progression and simplified the model to depict the causal process leading to prevalence of CIN2+ at a single point in time.

**Figure 2:**
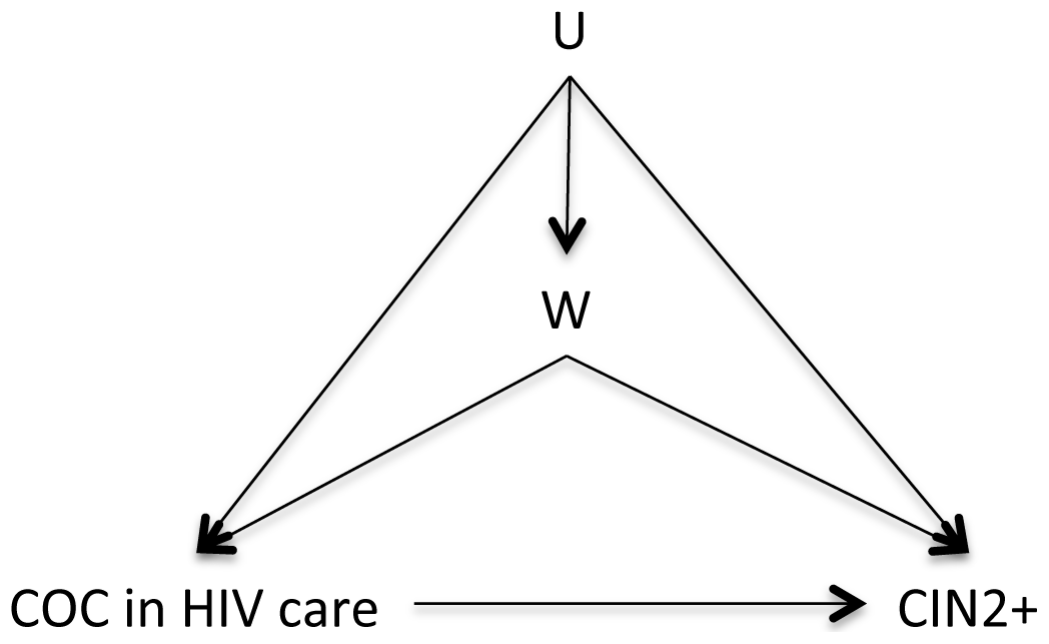
Reduced directed acyclic graph. Causal model reflecting observed data structure and assumptions required. COC: combined oral contraception; CIN2+: cervical intraepithelial neoplasia grades II and above.

In addition, the conceptual model has implications for covariate selection in estimation. Lifetime pregnancy experience is a time-dependent confounder; lacking longitudinal data, we chose to control for gravidity at enrollment in HIV care (gravidity at time = 1) as a proxy of past gravidity. In contrast, we did not control for recent condom use as it is not itself a confounder and is likely to have substantially more variability as a proxy for past condom use than baseline gravidity does for prior pregnancy experience. HIV treatment is a confounder; however it does not have to be part of a sufficient set of confounders if immune status is controlled for as a confounder. These decisions enabled us to considerably simplify the model, condensing all remaining confounders (age, educational attainment, marital status, gravidity, and immunosuppression) into a single node W and collapsing all unmeasured variability into the U node (Figure 2).

### Identifiability

The identifiability assumptions for the result to be a consistent estimate of the true causal relationship imply that all common causes of COC and CIN2+ must be measured; this is clearly violated given unmeasured covariates such as past COC use. Of the other 2 assumptions, unmeasured common causes of COC use and confounders are likely to exist, such as pregnancy intention for COC and gravidity. It is more plausible to assume that common causes of the confounders and CIN2+ are all measured, although data on duration of HIV for instance would strengthen this assumption. Nonetheless, this assumption alone is insufficient to infer causality. In sum, the identifiability assumptions are not met in these observed data, meaning that the estimate from these data will be confounded relative to the causal effect.

### Estimation

Of the 2519 women screened and eligible for inclusion, 219 were diagnosed with CIN2+; 89 of these cases were among 890 COC ever-users (10.00% prevalence) vs. 130 cases among 1629 non-users (7.98% prevalence). Regression analysis indicates the odds of CIN2+ are 1.35 times greater among ever-users of COC (95% CI 0.99, 1.85) than non-users after controlling for covariates. The g computation results suggest an adjusted prevalence difference of 2.5% (95% CI −0.2, 5.1) associated with ever using COC; estimates from IPTW and TMLE are slightly higher at 2.9%, and the confidence intervals around these estimates exclude 0 (Table 1). The consistency of the estimates suggests minimal model mis-specification from the use of a parametric model in the g computation estimate.

**Table 1:** Adjusted prevalence difference in CIN2+ between COC users and non-users.

	G computation	Inverse probability of treatment weighting	Targeted maximum likelihood estimation
**Main analysis**			
Estimate (95% CI)	0.025 (−0.002, 0.051)	0.029 (0.003, 0.056)	0.029 (0.001, 0.058)
Bootstrap mean (quantile-based 95% CI)	0.026 (0.001, 0.052)	0.030 (0.005, 0.055)	0.037 (0.008, 0.066)
**Sensitivity analysis**			
Estimate (95% CI)	0.003 (−0.032, 0.038)	0.019 (−0.018, 0.056)	0.022 (−0.028, 0.072)
Bootstrap mean (quantile-based 95% CI)	0.003 (−0.030, 0.036)	0.011 (−0.022, 0.045)	0.021 (−0.024, 0.076)

**Figure 3:**
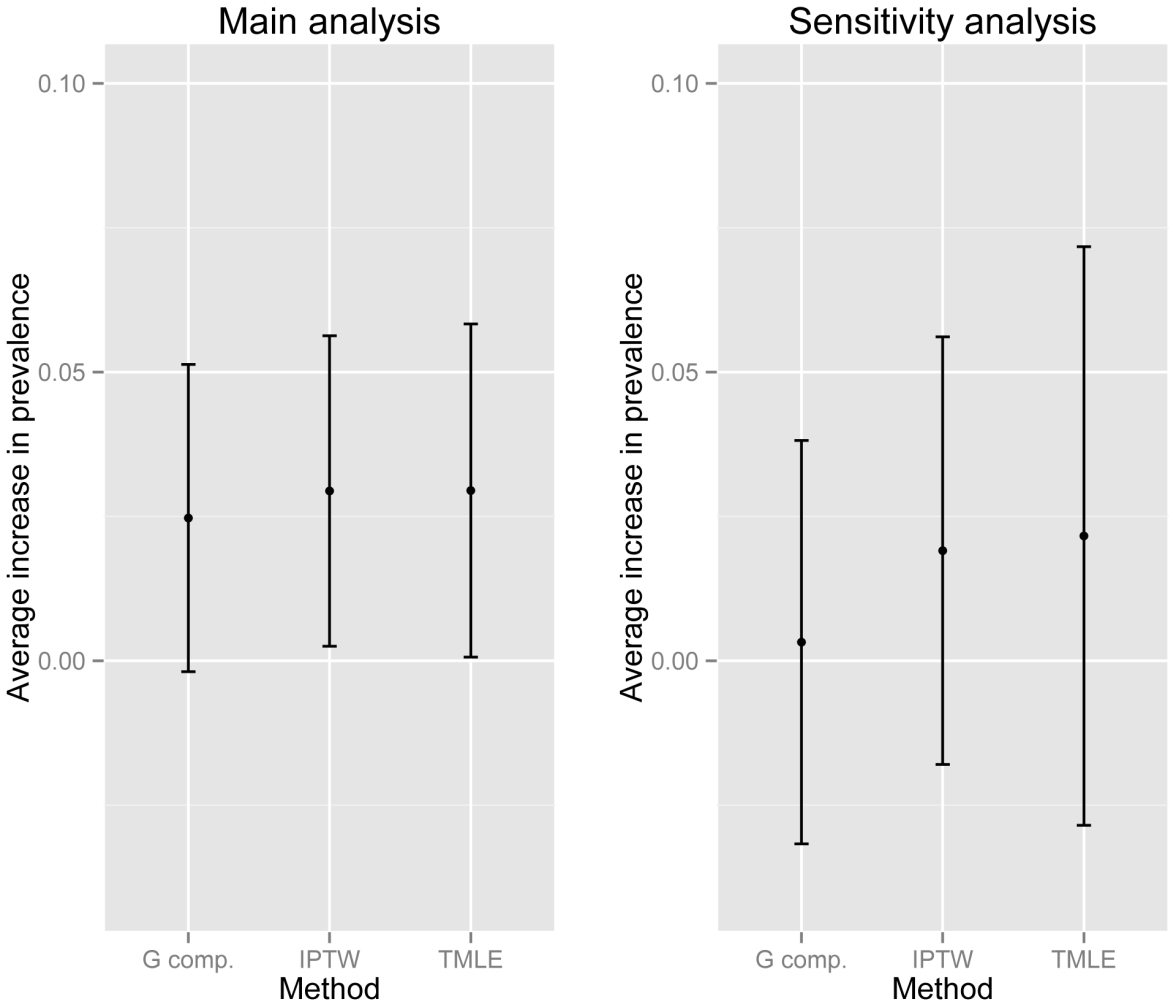
Difference in CIN2+ prevalence associated with COC. Predicted percent difference in prevalence of CIN2+ if all women were exposed to COC using 3 semi-parametric estimation methods. G comp: G computation; IPTW: Inverse probability of treatment weighting; TMLE: targeted maximum likelihood estimation; COC: combined oral contraception; CIN2+: cervical intraepithelial neoplasia grades II and above.

Table 1 and Figure 3 also show the sensitivity analysis results, which are less consistent; each estimate is smaller than the main analysis and none is statistically significant. Although there is no reason to believe exposure is a theoretical impossibility within any combination of covariate values, review of the data show practical positivity violations, such as that there are no women with greater than a secondary education who are unmarried, nulliparous, and use COC. However, the mean and quantile-based variance of the bootstrap samples in Table 1 show that these samples are reasonably symmetric and centered around the original estimate, indicating that the impact of near positivity violations is likely to be minimal. To draw valid inference despite missing data, we would need to restrict the target population to those represented in the sample or assume the observed association can be extrapolated to the unobserved groups [38].

## Discussion

The application of DAGs and semi-parametric estimation to the question of whether COC use increases the risk of cervical cancer among women with HIV demonstrates the conceptual and analytic benefits of a causal inference approach to observational data. Broader use of such tools can strengthen the quality of evidence considered for pressing public health questions by clarifying the question of interest, identifying critical variables required to estimate a causal quantity, and ensuring that estimation returns the quantity of interest without undue reliance on parametric model assumptions. In this example, over 35% of women in HIV care reported use of COC, a critical element of women's control of their reproduction that may be a carcinogen. Valid evidence of harm is required to implement sound public health policies for this vulnerable population. The causal model proposed for this research question codified the beliefs and hypotheses framing the analysis in a legible form that can be challenged and modified by other investigators. Further, the decisions made in developing that model have direct implications for the analysis: we did not control for covariates such as HAART status and condom use despite the fact that on first inspection they could be considered as confounders. DAGs provide more specific guidance for confounder inclusion than conceptual definitions of confounders alone and render visible the thought process behind inclusion and exclusion of covariates [18], [39]. In other examples, the full causal model may reveal multiple sufficient sets of confounders, enabling investigators to select based on pragmatic considerations of data collection and analysis.

DAGs elucidate the assumptions required for a causal effect to be estimable in observational data. Such guidance is particularly relevant for questions such as this that relate to complex and time-dependent interrelationships between behavior and biology. In this case, although 2 of the estimation methods employed suggest a statistically significant increase in prevalence of CIN2+ associated with ever using COC among HIV-positive women under 50 in Kenya, the identifying assumptions required to interpret this result as indicative of a causal relationship are untenable. They include: no reverse causation between exposure and covariates selected for control; no remaining common causes of recent COC use and CIN2+ following control for age, education, marital status, gravidity, and CD4+ cell count nadir; and no remaining causes of either COC and the confounders or CIN2+ and the confounders. Specific unmeasured covariates such as past sexual behavior render key assumptions implausible. It is possible to postulate the direction of bias due to individual confounders: for example, multiple sexual partnerships would positively confound the relationship between COC and CIN2+ due to its positive association with each. However, the number of unmeasured or unknown covariates makes estimating their joint impact difficult and beyond the scope of this analysis (see reference [37] for discussion of multivariate bias analysis).

Consideration of identifiability also guides prioritization of future data collection: it may be easier to satisfy the assumption that all common causes of the outcome and covariates are measured, making it unnecessary to also measure all common causes of exposure and covariates. The granularity of causal assumption checking provides guidance for future research studies. In this application, collection of longitudinal or retrospective data on COC use is critical to isolating causal pathways of interest. More refined data on COC use would also address bias due to exposure misclassification in this analysis, which appears likely based on the change in results seen in the sensitivity analysis using a stricter classification of exposure. In addition, collecting data on covariates such as duration of HIV may be more useful than quantifying challenging constructs such as pregnancy intentions over time in terms of satisfying the assumptions to infer causality from observed data.

The 3 semi-parametric estimation approaches presented are a natural extension of the conceptual approach. In comparison to a traditional regression, which provides a prevalence odds ratio conditional on covariates, the tools applied enable estimation of a quantity of key public health interest: how much the prevalence of CIN2+ would change if COC use were halted or if family planning programs and contraceptive use achieved a wider coverage among HIV-infected women. These approaches provide the single quantity of interest defined in the causal model. In contrast, multivariate regression returns coefficients for the exposure of interest and for confounders; the latter can be misinterpreted as representing causal relationships [40]. Further, although g computation, IPTW, and TMLE can be fit using parametric regression models, an additional benefit is the potential to combine them with model fitting procedures to reduce bias. G computation is vulnerable to bias if the model for the outcome is incorrect, while IPTW requires the treatment mechanism be correct. TMLE is consistent if either the treatment mechanism or outcome expectation is correctly estimated and provides theoretically valid variance estimates even when employing automated model fitting. It can be implemented using standard software to reduce the dependence of estimates on model form. The availability of multiple estimation options permits selection of the tool that may be best supported by the observed data, such as IPTW if estimation of the process leading to exposure is considered more reliable than estimation of the outcome or TMLE in the case where either exposure or outcome estimation is thought to be consistent. Although not applied here, extensions of these methods enable estimation of more flexible and realistic parameters, such as the effect on CIN2+ prevalence if 50% of women used COC or only those women with high CD4+ count used COC [41].

Taking a structured approach to developing a causal question is a core practice of public health researchers; the particular approach demonstrated here provides a systematic and transparent method for framing the question, depicting assumptions transparently, estimating the parameter, and drawing inference. Application of causal inference methods such as these can enrich observational epidemiologic studies by improving the clarity of communication around causal hypotheses, providing theory-driven guidance on confounder selection, and matching the estimation method to the question of interest without undue reliance on correct model specification. Determining whether COC use may increase cervical cancer risk is critically important, particularly given that the approximately 17 million women currently living with HIV [42], [43] may face an elevated baseline risk of cervical cancer. Utilizing a causal framework and analytic methods can provide clear guidance on the remaining research gaps that must be addressed to answer this question, and many others.

## Supporting Information

Figure S1 Reading directed acyclic graphs. An estimate will reflect a true causal relationship (be unconfounded) if a set of measured variables fulfills the backdoor criterion: the set contains no variable caused by X, and, after conditioning on all variables in the set, all paths connecting X to Y that include an arrow into X are blocked by either a conditioning variable or a variable where 2 arrows collide. The backdoor criterion can be read off of DAGs following the rules demonstrated here. **Panel A**. - X may cause Y; W may cause X and Y; there are unknown causes of X (U_X_), Y (U_Y_), and W (U_W_). - U_X_ does not cause W. In other words, although there are unknown causes of both X and W, there are no shared causes of these variables. U_X_ and U_W_ are independent. - X and Y will be associated in observed data under the null hypothesis of no effect of X on Y. The biasing pathway X – W – Y is called a backdoor path because it starts with an arrow pointing to X. Controlling for W renders X and Y unassociated except for any direct effect between them. - Sufficient set of confounders: {W}. **Panel B**. - X and Y will be unassociated in observed data under the null hypothesis of no effect of X on Y. The path X-W-Y is closed due to the paths from X and Y colliding at W; no association travels along this path. If this is the complete causal structure, controlling for W creates an association between X and Y that will bias any true causal effect. Conditioning on colliders opens the path the collider is on and should be avoided whenever possible. - Sufficient set of confounders: {}. **Panel C**. - X – W3 – Y is an open backdoor path that will bias the X-Y association. - Controlling for W3 opens the path X – W1 – W2 – Y, introducing a new bias. - Either W1 or W2 blocks the new path. - Sufficient set of confounders: {W1, W3}, {W2, W3}.
(TIF)
