# Supplementary figures and images for: Cervical Cancer Precursors and Hormonal Contraceptive Use in HIV-Positive Women: Application of a Causal Model and Semi-Parametric Estimation Methods
Source: PLoS One. 2014 Jun 30;9(6):e101090. doi: 10.1371/journal.pone.0101090 (PMC4076246; doi:10.1371/journal.pone.0101090)

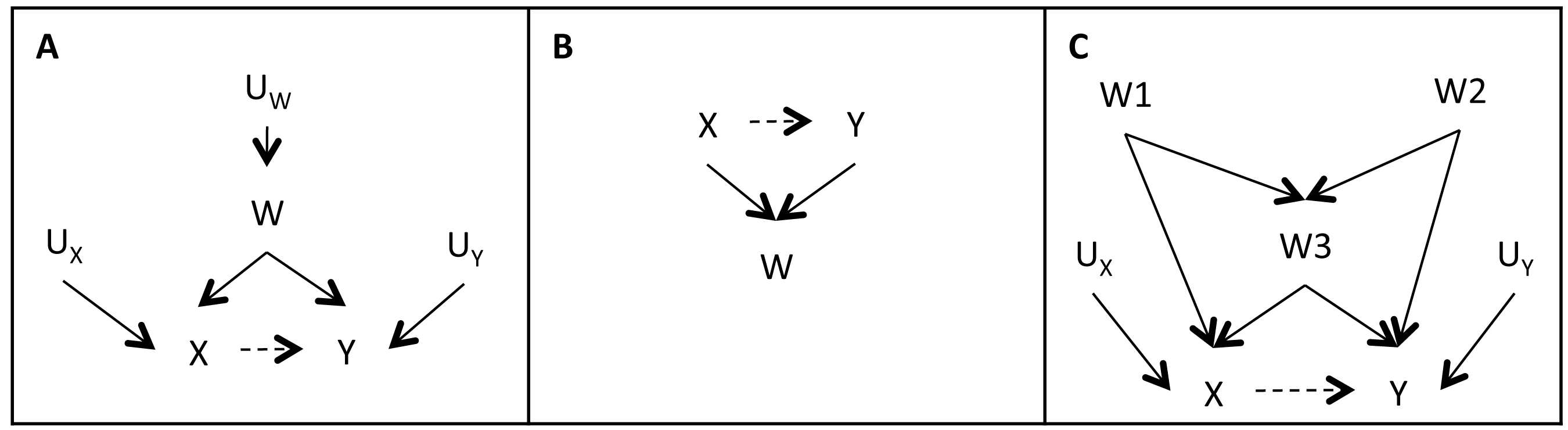

Supplement: Figure S1 — Reading directed acyclic graphs. An estimate will reflect a true causal relationship (be unconfounded) if a set of measured variables fulfills the backdoor criterion: the set contains no variable caused by X, and, after conditioning on all variables in the set, all paths connecting X to Y that include an arrow into X are blocked by either a conditioning variable or a variable where 2 arrows collide. The backdoor criterion can be read off of DAGs following the rules demonstrated here. Panel A. - X may cause Y; W may cause X and Y; there are unknown causes of X (UX), Y (UY), and W (UW). - UX does not cause W. In other words, although there are unknown causes of both X and W, there are no shared causes of these variables. UX and UW are independent. - X and Y will be associated in observed data under the null hypothesis of no effect of X on Y. The biasing pathway X – W – Y is called a backdoor path because it starts with an arrow pointing to X. Controlling for W renders X and Y unassociated except for any direct effect between them. - Sufficient set of confounders: {W}. Panel B. - X and Y will be unassociated in observed data under the null hypothesis of no effect of X on Y. The path X-W-Y is closed due to the paths from X and Y colliding at W; no association travels along this path. If this is the complete causal structure, controlling for W creates an association between X and Y that will bias any true causal effect. Conditioning on colliders opens the path the collider is on and should be avoided whenever possible. - Sufficient set of confounders: {}. Panel C. - X – W3 – Y is an open backdoor path that will bias the X-Y association. - Controlling for W3 opens the path X – W1 – W2 – Y, introducing a new bias. - Either W1 or W2 blocks the new path. - Sufficient set of confounders: {W1, W3}, {W2, W3}. (TIF) [file pone.0101090.s001.tif]
